# Psychiatric Comorbidities and Quality of Life in Patients with Vestibular Migraine and Migraine without Vertigo: A Cross-Sectional Study from a Tertiary Clinic

**DOI:** 10.3390/audiolres14050065

**Published:** 2024-09-05

**Authors:** Franko Batinović, Davor Sunara, Vana Košta, Milena Pernat, Tonći Mastelić, Ivan Paladin, Nikolina Pleić, Jure Krstulović, Zoran Đogaš

**Affiliations:** 1 Department of Otorhinolaryngology, University Hospital of Split, Spinčićeva 1, 21000 Split, Croatia; fbatinovic1@gmail.com (F.B.); davor.sunara@gmail.com (D.S.); ivan.paladin@gmail.com (I.P.); 2 Department of Neurology, University Hospital of Split, Spinčićeva 1, 21000 Split, Croatia; vanakosta@gmail.com; 3 Department of Psychiatry, University Hospital of Split, Spinčićeva 1, 21000 Split, Croatia; milenapernat02@gmail.com (M.P.); toncimastelic@hotmail.com (T.M.); 4 Department of Biology and Human Genetics, School of Medicine, University of Split, Šoltanska 2, 21000 Split, Croatia; 5 Department of Computer Engineering, University of Applied Sciences ASPIRA, Domovinskog rata 65, 21000 Split, Croatia; 6 Department of Health Care Quality, University Hospital of Split, Spinčićeva 1, 21000 Split, Croatia; jure.krstulovic@gmail.com; 7 Department of Neuroscience and Sleep Medicine Center, School of Medicine, University of Split, Šoltanska 2, 21000 Split, Croatia; zdogas@gmail.com

**Keywords:** vestibular migraine, migraine, vertigo, anxiety, depression

## Abstract

Background Clinical studies suggest that vestibular migraine patients have psychiatric comorbidities and low life quality. However, the absence of a multidisciplinary approach to vestibular migraine patients, including otorhinolaryngologists and psychiatrists, is concerning. We aimed to investigate these patients comprehensively and to compare the results of three questionnaires—the Hospital Anxiety and Depression Scale (HADS), Dizziness Handicap Inventory (DHI), and Short Form Health Survey (SF-36)—between patients with definite vestibular migraine (dVM), migraine without vertigo (MO), and healthy controls (HCs). Methods: A total of 104 participants were divided into 3 groups: dVM patients (19 participants), MO patients (22 participants), and HCs (63 participants). The scores of the three questionnaires across the three groups were compared using analysis of variance, and linear regression was used to examine the associations between the questionnaire scores within each group. Results: Compared to MO patients and HCs, dVM patients had significantly higher total scores on the HADS (*p* < 0.0001) and DHI (*p* < 0.0001) scales, and lower scores for all nine components of the SF-36, indicating poorer health. In the vestibular migraine group, the DHI score was strongly negatively correlated with the Physical Functioning subscale of the SF-36. Conclusions: Anxiety and depression are more prevalent in patients with definite vestibular migraine compared to patients with migraine without vertigo and healthy controls. The physical functioning of patients with definite vestibular migraine is highly affected by their dizziness, resulting in a lower quality of life. Timely screening for psychiatric comorbidity in vestibular migraine patients is essential to prevent psychiatric consequences.

## 1. Introduction

Vestibular migraine (VM) is a common cause of episodic vertigo where migraine headaches and vestibular dysfunction overlap and co-exist [1]. VM affects approximately 1% of the general population and 10% of migraine patients [2,3]. The exact pathophysiology is still unclear [2,3,4].

There are two types of VM—probable and definite VM (dVM) [5]. According to the jointly elaborated diagnostic criteria for dVM by the International Classification of Headache Disorders (ICHD) and the Barany Society [5,6], the duration of an attack can vary from 5 min to 72 h. Furthermore, the vestibular symptoms should appear at least five times in a lifetime without being explained by other diagnoses [4,5]. During at least 50% of vertigo episodes, patients need to exhibit one or more migraine features, such as unilateral and pulsating headaches aggravated by routine physical activity [1,5,6]. There are no medical tests that are pathognomonic for dVM patients [7], and diagnosis is typically based on the patient’s clinical history and the exclusion of other vestibulopathy [1,4].

Similar pathophysiological pathways intertwine between the vestibular and psychiatric systems [8]. The thalamocortical circuit, cerebellar, and limbic pathways are involved in a complex network that connects the vestibular and psychiatric domains [8]. It has been proposed that the co-occurrence of anxiety, vertigo, and migraine disorders is a single monoaminergic pathway disorder called migraine-related dizziness (MARD) [9]. Compared to patients without psychiatric comorbidity, MARD patients respond poorly to migraine therapy (also used for VM) [9,10]. Patients with vertigo and psychiatric comorbidities experience a lower quality of life and frequently use the healthcare system services compared to those without any psychiatric comorbidity [11]. To improve the mental health of VM patients and reduce unnecessary healthcare costs, it is essential to identify and treat these patients in a timely manner [12]. This study demonstrates a multidisciplinary approach to dVM patients involving clinical examinations by a neurologist, psychiatrist, and otorhinolaryngologist. The Dizziness Handicap Inventory (DHI) and the Short Form Health Survey (SF-36) are reliable indicators of how vertigo impacts life quality in patients with vertigo [13,14,15].

To date, this is the first study to compare the results of the SF-36, DHI, and Hospital Anxiety and Depression Scale (HADS) and their subscales between dVM patients, patients with migraine without vertigo (MO), and healthy controls (HCs). Therefore, this study aimed to identify the most common psychiatric comorbidities in patients with dVM through clinical-based psychiatric examination and HADS scales. This study will provide insights into the questionnaire results on the dizziness handicap, quality of life, and mental health of dVM patients compared to MO patients and HCs.

## 2. Materials and Methods

### 2.1. Study Design

**Figure 1:**
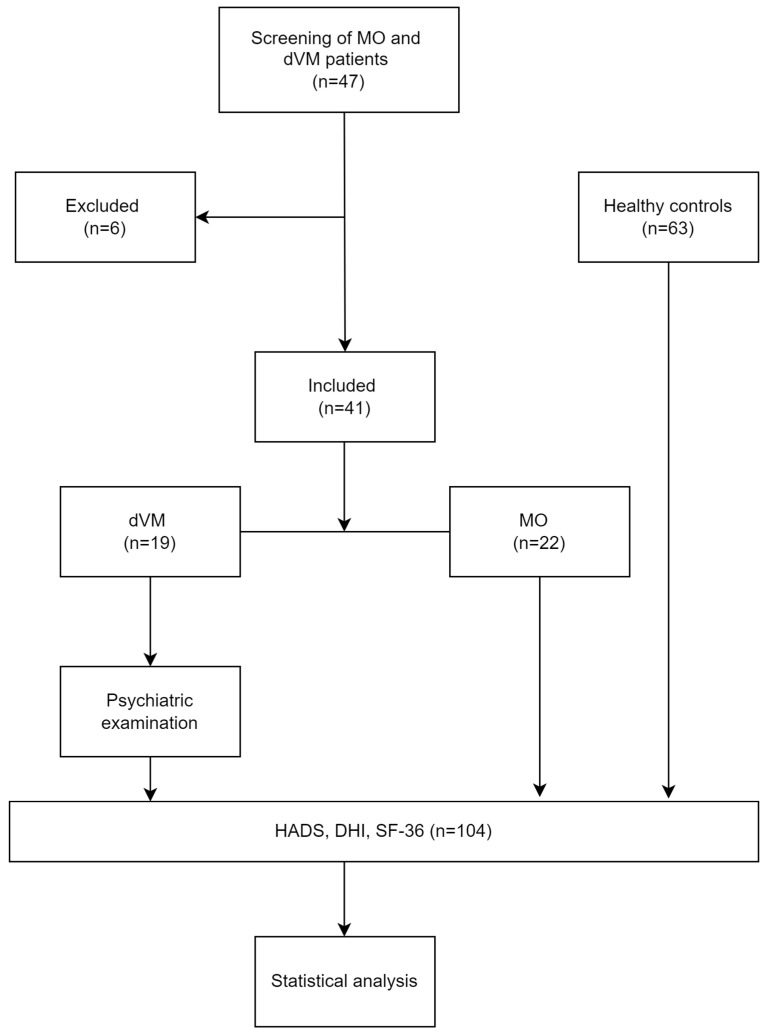
Flowchart of participant screening and group allocation.

This cross-sectional study was conducted between May 2022 and May 2023 at the Otorhinolaryngology tertiary clinic, University Hospital of Split, Croatia. All patients with a diagnosis of migraine and VM who arrived at the Emergency Department in an acute attack were examined by a neurologist and otorhinolaryngologist (Figure 1). Ethical approval was provided by the Ethical Committee of the University Hospital Split (No. 2181-147-01/06/M.S.-21-02). Our research methods and reporting align with the STROBE guidelines, as detailed in Supplementary File S3.

### 2.2. Participants

We included participants over 18 years of age with a confirmed diagnosis of migraine or dVM according to the ICHD and Barany Society criteria [5,6]. The HCs without any diagnosis associated with migraine or vertigo were also included. We excluded the following: (i) patients with other audio-vestibular disorders and functional dizziness; (ii) patients with other neurological diseases; (iii) participants with recorded drug or alcohol abuse; (iv) VM or MO patients with diagnosed psychiatric disorders who did not take the prescribed therapy for the last six months.

All patients were examined according to the ICHD and Barany’s criteria implemented in the checklists (Supplementary File S2). The oto-neurological examination of dVM patients included otoscopy, the HINTS battery, the STANDING algorithm, the oculomotor examination with spontaneous nystagmus (in primary gaze, gaze holding, and with Frenzel’s glasses), positional testing (Dix–Hallpike and lateral roll tests), truncal ataxia, and the House Brackmann score [16].

The otorhinolaryngologist examined the vestibulo-ocular reflex (VOR) of six semicircular canals via video head impulse test (vHIT; GN Otometrics, Taastrup, Denmark 2019) in an acute period of all dVM patients. The *HINTS* examination enhanced by vHIT recording is superior to the bedside HINTS examination in patients suffering from acute vestibular syndrome [17]. For details of measurements, see [17]. The function of the otolith organs was examined with subjective visual vertical (SVV) by the same otorhinolaryngologist in all dVM patients. SVV is an effective technique for assessing the function of central vestibular pathways [18]. We performed the measurement technique described by Chang TP. et al. [18]. The hearing levels were documented by pure tone audiometry mainly to differentiate between VM, sudden hearing loss, and Meniere’s disease [19]. All patients underwent 1.5 Tesla magnetic resonance imaging (MRI) of the brain evaluated by a neuroradiologist. The same senior psychiatrist examined all dVM patients using the structured clinical interview in the symptom-free phases. The psychiatric diagnoses were based on the Diagnostic and Statistical Manual 5 of Mental Disorders criteria (DSM-5), the gold standard for diagnosing mental disorders [7,20], and the HADS scale. Questionnaires were administered to dVM patients after they had received symptomatic treatment and the clinician had confirmed the post-ictal period of vestibular migraine. Additionally, a key strength of this approach is that all patients completed the questionnaire at a consistent time point after receiving the same symptomatic medications (granisetron and metamizole). This standardized timing minimizes variability, ensuring that patients were not at home using different treatments independently before filling out the questionnaires. All participants (dVM, MO, and HCs) provided written informed consent (Supplementary File S1) and completed the HADS, DHI, and SF-36 questionnaires.

### 2.3. Questionnaires

The HADS is a fourteen-item scale with seven sections for anxiety and depression subscales. Scoring for each item ranges from 0 to 3 [21]. A subscale score ≥ 8 denotes anxiety or depression [22,23]. We utilized this tool to anticipate patients’ psychiatric comorbidity, including anxiety and depression.

The DHI scale consists of 25 questions that assess the extent of impairment in patients with vertigo based on emotional, functional, and physical aspects. Whitney’s grading standards categorize patients with a score of 0–30 as having minor disorders, 31–60 as moderate, and 61–100 as severe disorders [24].

The SF-36 consists of 36 ordinal scale items designed to assess health-related quality of life in physical and psychological concepts. Thirty-five of these items are used to construct eight different scales (“Physical functioning”, “Role limitations due to physical health”, “Bodily pain”, “General health”, “Vitality”, “Social functioning”, “Role limitations due to emotional problems”, and “Mental health”), while the remaining item evaluates “Health transition” [25,26]. All questionnaires were translated, validated, and already used in Croatian studies [13,23,25,26].

### 2.4. Statistical Analysis

Continuous variables are presented as means with standard deviations or medians with minimum and maximum values, while categorical variables are presented as absolute frequencies (relative frequencies). The χ2-test was used to assess the differences between groups for categorical variables, and ANOVA or linear regression was used for continuous variables. The associations between HADS, DHI, and SF-36 scores were tested using regression models adjusted for known confirmed confounders of age and sex. Regression assumptions, including the linearity of the data, normality of residuals, and homoscedasticity, were checked using diagnostic plots. To control for multiple comparisons and reduce the risk of Type I errors, the *p*-values were adjusted using the Benjamini–Hochberg procedure, which controls the False Discovery Rate (FDR). Statistical analyses were conducted using *R* software version 4.1.3 [27], with a significance level of 0.05.

## 3. Results

### 3.1. Demographic Characteristics of Participants

Table 1 presents the demographic and clinical characteristics of dVM patients, MO patients, and HCs. The study included 19 patients with dVM, consisting of 16 women and 3 men, with a mean age of 48 years (SD: 14.3, age range: 25–70 years). Additionally, 22 MO patients were involved, including 20 women and 2 men, with an average age of 41 years (SD: 13.5, age range: 22–75 years). The study also encompassed 63 HCs, consisting of 44 women and 19 men, with an average age of 28 years (SD: 12.1, age range: 20–63 years). Healthy controls were notably younger than VM and MO patients (Table 1, *p* < 0.0001 and *p* = 0.011, respectively). Because of this, the difference in questionnaire scores between the groups was tested using linear regression, with age included as an additional independent variable to control for its confounding effect. Conversely, there was no significant difference in age between the dVM and MO patient groups (Table 1, *p* = 0.276). Furthermore, there was no significant difference in the sex distribution across the three groups (Table 1, *p* = 0.092).

### 3.2. Comparison of HADS Scores between the Groups

The mean total HADS score for dVM patients was 17.6 (SD: 4.99; range: 9–27). Specifically, the mean score for the HADS-A subscale in dVM patients was 9.37 (SD: 2.27; range: 6–14), and for the HADS-D subscale, it was 8.26 (SD: 3.05; range: 3–13). Based on the HADS-A and HADS-D subscale scores ≥ 8, 13 out of 19 dVM patients (68.42%) exhibited indications of anxiety, and 11 out of 19 (57.89%) showed signs of depression. The results from HADS-A follow psychiatrists’ clinical diagnoses, where 11 out of 19 (57.89%) dVM patients were diagnosed with anxiety. The prevalence of diagnosed depression was, however, lower, with only one (5.26%) dVM patient diagnosed with depression by a psychiatry specialist.

The mean total HADS score for MO patients was 10.8 (SD: 4.66; range: 3–21). Specifically, the mean score for the HADS-A subscale in MO patients was 6.50 (SD: 2.50; range: 2–11), and for the HADS-D subscale, it was 4.32 (SD: 2.71; range: 0–10). Based on the HADS-A and HADS-D subscale scores ≥ 8, 9 out of 22 MO patients (40.91%) exhibited signs of anxiety, and 4 out of 22 (18.18%) showed indications of depression. The prevalence of anxiety based on HADS-A results was not significantly different between the dVM and the MO group of patients (χ^2^ = 3.103, *p* = 0.078). The prevalence of depression based on HADS-D results was significantly higher in the dVM compared to the MO group of patients (χ^2^ = 6.930, *p* = 0.008).

The mean total HADS score for HCs was 8.16 (SD: 4.25; range: 0–18). Specifically, the mean score for the HADS-A subscale in HCs was 5.08 (SD: 2.55; range: 0–10), and for the HADS-D subscale, it was 3.08 (SD: 2.43; range: 0–10). Based on the HADS-A and HADS-D subscale scores ≥ 8, 13 out of 63 HCs (20.63%) exhibited indications of anxiety, and 4 out of 63 (6.35%) showed indications of depression. The prevalence of anxiety based on HADS-A results was significantly different between the dVM and the HC group (χ^2^= 15.394, *p* = 0.00009), but not between the MO and the HC group (χ^2^= 3.494, *p* = 0. 062). The prevalence of depression based on HADS-D results was significantly different between the dVM and the HC group (χ^2^ = 25.949, *p* < 0.00001), but not between the MO and the HC group (χ^2^ = 2.678, *p* = 0.102).

The total HADS score differed significantly across the three groups (Table 1, *p* < 0.0001). Patients with dVM had significantly higher total HADS scores compared to MO patients (Table 1, *p* < 0.0001) and compared to HCs (Table 1, *p* < 0.0001). Additionally, there was a statistically significant difference in the HADS-D subscale score across groups (Table 1, *p* < 0.0001). Patients with dVM had significantly higher HADS-D scores compared to MO patients (Table 1, *p* < 0.0001) and compared to HCs (Table 1, *p* < 0.0001). Finally, there was a statistically significant difference in the HADS-A subscale score across groups (Table 1, *p* < 0.0001). The dVM patients had significantly higher HADS-A scores compared to MO patients (Table 1, *p* = 0.001) and compared to HCs (Table 1, *p* < 0.0001).

### 3.3. Comparison of DHI Scores between the Groups

The mean DHI score for dVM patients was 37.6 (SD: 16.1; range: 14–86), which, according to Whitney’s grading standards, classifies these patients as having a moderate handicap on average. The mean DHI score for MO patients was 18 (SD: 17.2; range: 0–60), reflecting a mild handicap on average. Healthy controls had a mean DHI score of 2.32 (SD: 5.99; range: 0–32). The DHI score differed significantly across the three groups (Table 1, *p* < 0.0001). Patients with dVM had significantly higher DHI scores compared to MO patients (Table 1, *p* < 0.0001) and compared to HCs (Table 1, *p* < 0.0001). The DHI score additionally differed significantly between the MO patients and HCs, with MO patients having higher DHI scores (Table 1, *p* < 0.0001).

### 3.4. Comparison of SF-36 Scores between the Groups

Each of the nine components of the SF-36 questionnaire differed significantly between the three groups (Table 2). SF-36 items were recoded so that high scores indicate good health. Patients with dVM had substantially lower scores for each of the nine components: Physical functioning, Role-Physical, Bodily Pain, General Health, Vitality, Social Functioning, Role-Emotional, Mental Health and Health Transition compared to MO patients and HCs. Additionally, MO patients had significantly lower scores for five out of the nine components: Physical functioning, Role-Physical, Bodily Pain, General Health, and Social Functioning, compared to HCs (Table 2).

### 3.5. Correlation of Different Questionnaire Scores in Each Group

In the dVM group, the DHI score was strongly negatively correlated with the Physical functioning subscale of the SF-36 (β = −0.862, *p* < 0.001). All SF-36 subscales, except Health Transition, showed negative correlations with HADS, HADS-A, HADS-D, and DHI scores, although these correlations were not statistically significant. In the MO group, the total HADS score was significantly negatively correlated with the Role-Physical, General Health, and Social Functioning subscales (Table 3). The HADS-A score showed significant negative correlations with the General Health and Mental Health subscales. The HADS-D score was significantly negatively correlated with the Role-Physical, General Health, and Vitality subscales of the SF-36. Although all SF-36 subscales, except for Health Transition, demonstrated negative correlations with the HADS, HADS-A, HADS-D, and DHI scores, these correlations were not statistically significant. The HCs’ total HADS score showed significant negative correlations with the Role-Physical, Bodily Pain, General Health, Vitality, Social Functioning, Role-Emotional, and Mental Health subscales of the SF-36. The HADS-A score was significantly negatively correlated with the Bodily Pain, General Health, Vitality, Social Functioning, and Mental Health subscales. The HADS-D score exhibited significant negative correlations with the Role-Physical, General Health, Vitality, and Social Functioning subscales. All SF-36 subscales showed negative correlations with HADS, HADS-A and HADS-D, although these correlations were not statistically significant. Additionally, the DHI score was positively correlated with the total HADS score, HADS-A, and HADS-D, and negatively correlated with the Bodily Pain, General Health, Vitality, Social Functioning, Role-Emotional, Mental Health, and Health Transition subscales.

## 4. Discussion

Our study highlighted significant differences in HADS and DHI scores and SF-36 subscale scores, between dVM, MO, and HC groups. Patients with dVM had significantly higher total HADS, HADS-A, and HADS-D subscale scores than MO patients and HCs. Additionally, dVM patients exhibited significantly higher DHI scores and lower scores across all SF-36 subscales compared to the other groups. MO patients also showed significantly lower scores in several SF-36 subscales than HCs. Regarding correlations, in the dVM group, the DHI score was strongly negatively correlated with the Physical functioning subscale of the SF-36. Almost all SF-36 subscales showed negative correlations with the HADS, HADS-A, HADS-D, and DHI scores in all three groups. However, these correlations were not all statistically significant.

Since ancient times, there has been a fundamental interaction between the vestibular and psychiatric systems [28]. Psychiatric comorbidities like anxiety, phobic disorders, and depression are commonly found in patients with vertigo in clinical studies [12,29,30,31,32,33]. Additionally, MO patients have significantly higher rates of psychiatric comorbidities compared to the general population [34]. A meta-analysis from 2023 demonstrated that treatments with antidepressant effects, such as venlafaxine, effectively prevented VM attacks [10]. Our research and another cross-sectional study may support the findings that VM patients are more anxious than MO patients or HCs [35]. However, clinical data on psychiatric comorbidity in dVM patients, diagnosed by a psychiatrist according to DSM-5 criteria, are scarce.

Only two cross-sectional studies performed clinical psychiatric examinations according to DSM criteria in VM patients [12,35]. However, both studies used Neuhauser’s criteria for diagnosing VM [36] where two vertigo attacks were sufficient for VM diagnosis. Additionally, previous studies failed to consult an otorhinolaryngologist during the clinical examination of VM patients, which is essential to exclude conditions such as BPPV, Meniere’s disease, and ear infections. Furthermore, in our study, a senior psychiatrist conducted clinical examinations of dVM patients using the DSM-5 criteria [7,20]. In other studies, a general practitioner or interviewer diagnosed psychiatric comorbidities in VM patients with validated questionnaires [7,15,24,37,38]. We hypothesized that anxiety is the most common comorbidity in dVM patients based on clinical examination and the results of the HADS scores, which was confirmed by our analysis.

It is well known that VM patients have a higher incidence of anxiety, phobias, and depression compared to other vertigo subgroups [12,29]. Some authors have proposed that VM is strongly linked to psychiatric comorbidities due to the stressful coexistence of vestibular dysfunction and migraine headaches [3,7]. Three observational studies found that patients with VM were more prone to anxiety than depression [7,12,35], which is in line with our research. Minen et al. showed that about 50% of MO patients had anxiety, and about 40% had depression [34]. We found that 68.42% of dVM patients showed signs of anxiety, and 57.89% showed indications of depression. Furthermore, dVM patients were more depressed than MO patients according to the HADS-D scales.

Patients with vertigo have a lower life quality [2,3]. Only three clinical studies used the SF-36 questionnaire to assess the quality of life in dVM patients [14,15,39]. In two clinical studies, all subscale scores of the SF-36 were generally low for dVM patients [14,15]. Here, we showed that the majority of dVM patients who are clinically diagnosed with anxiety had lower scores across all SF-36 subscales compared to MO and HCs. Consequently, oto-neurological examination should include a psychiatric evaluation to prevent deterioration in the quality of life.

Several studies showed high DHI scores in patients with VM [15,24,38]. Balci et al. and Ak et al. [15,38] found that DHI sub-scores were significantly higher in VM patients than in HCs, which is consistent with our study. We also found that dVM patients had substantially higher DHI scores than MO patients. In contrast, Kim et al. showed that DHI scores did not correlate with daily vertigo symptoms in patients with VM [40]. Zhu et al. found that DHI scores in VM patients positively correlated with changes in anxiety and depression in the HADS score [24]. We also noted a positive correlation between DHI and HADS scores in VM patients. This suggests that it is important to place more emphasis on the mental health of VM patients as their dizziness handicap worsens.

The DHI and SF-36 subscales exhibit negative correlations [13,14]. We showed that the DHI score in dVM patients was strongly negatively correlated with the Physical functioning subscale of the SF-36. Ak et al. noted that all DHI subscores were significantly higher, and all SF-36 subscales were substantially lower in dVM patients than HCs [15]. In our study, dVM patients had considerably lower scores for each of the SF-36 components compared to MO patients and HCs. Considering the evidence, we emphasize that VM patients need to be examined in a timely and multidisciplinary manner (otorhinolaryngologist, neurologist, and psychiatrist) to prevent a reduction in quality of life and degradation of mental health.

Our study has many strengths. It is the first to examine dVM patients for psychiatric comorbidities according to the DSM-5. We compared the SF-36, DHI, and HADS scores and their subscales, between dVM patients, MO patients, and HCs. We only included patients diagnosed by Barany’s and ICHD criteria and examined by the same three clinicians (psychiatrist, otorhinolaryngologist, and neurologist) within 72 h after an acute attack. Our study highlighted the importance of a comprehensive clinical approach to dVM patients to differentiate organic and non-organic vertigo easily, providing a more objective three-dimensional picture of this multifactorial disorder. Furthermore, to exclude other oto-neurological pathology, we administered a detailed checklist following ICHD criteria, conducted PTA, VHIT, and SVV measurements, and performed brain MRI scans. Above all, we made pre-registrations in the Open Science Framework “https://archive.org/details/osf-registrations-zbwmq-v1, (accessed on 27 April 2022)” and determined the sample size before collecting VM patients.

Our study has several limitations. It is a single-center and cross-sectional study without data on patients’ follow-ups. We utilized a convenience sample of patients admitted to our clinic, which determined the sample size for this study. Also, we began recruiting VM patients at the end of the COVID-19 pandemic which has had psychiatric repercussions on the entire population, including migraine patients [41]. Furthermore, the SF-36, HADS, and DHI questionnaire results should be interpreted cautiously because they may reflect a subjective picture of the patient’s condition. Finally, the patient’s medical history was based on subjective reports. We addressed validity issues due to instrumentation by having every patient fill out a checklist and be examined by the same clinicians who created the checklist. It is important to note that the psychiatric clinical examination was performed after an acute VM attack, which may have led to false positive results. We suggest that future studies compare dVM patients with and without psychiatric comorbidities using the Vestibular Migraine Patient Assessment Tool and Handicap Inventory which provides more information about cognition and specific VM symptoms [40]. Additionally, it would be valuable to investigate the emotional, functional, and physical subscales of the DHI.

## 5. Conclusions

We effectively demonstrated the importance of timely and multidisciplinary clinical examination of dVM patients. Our findings highlight the value of distinguishing between different subgroups of migraine in comparative research, as psychiatric disorders may affect VM and MO patients differently. The majority of dVM patients were newly diagnosed with anxiety and were more depressed than MO patients. For the first time, we showed that dVM patients with psychiatric comorbidities have a lower quality of life than MO patients and HCs. This study could significantly improve the prevention of impairment in the quality of life and mental health of dVM patients by requesting psychiatric examinations in the post-ictal period.

## Figures and Tables

**Table 1:** Demographic and clinical characteristics of participants.

	Definite Vestibular Migraine (dVM) *n* = 19	Migraine (MO) *n* = 22	Healthy Controls (HC) *n* = 63	*p*-Value	Pairwise Comparisons	
					Comparison	*p*-Value
Age (years)	50 (25–70)	41 (22–75)	28 (20–63)	**<0.0001** **^a^**	VM-MO	0.276
					VM-HC	**<0.0001**
					MO-HC	**0.011**
Female	16 (84.21%)	20 (90.91%)	44 (69.84%)	0.092 ^b^	-	-
HADS	17.6 (4.99)	10.8 (4.66)	8.16 (4.25)	**<0.0001** **^c^**	VM-MO	**<0.0001**
					VM-HC	**<0.0001**
					MO-HC	0.065
HADS-A	9.37 (2.27)	6.5 (2.50)	5.08 (2.55)	**<0.0001** **^c^**	VM-MO	0.0007
					VM-HC	**<0.0001**
					MO-HC	0.047
HADS-D	8.26 (3.05)	4.32 (2.71)	3.08 (2.43)	**<0.0001** **^c^**	VM-MO	**<0.0001**
					VM-HC	**<0.0001**
					MO-HC	0.207
DHI	37.6 (16.1)	18 (17.2)	2.32 (5.99)	**<0.0001** **^c^**	VM-MO	**<0.0001**
					VM-HC	**<0.0001**
					MO-HC	**<0.0001**

Variables are represented as median (minimum-maximum), mean (SD) or absolute frequency (relative frequency). ^a^ ANOVA test, ^b^ χ^2^ test, ^c^ linear regression with age as an additional independent variable. *p*-values marked in bold indicate statistically significant differences between the groups. DHI, The Dizziness Handicap Inventory; HADS, Hospital Anxiety and Depression Scale.

**Table 2:** SF-36 component distributions across groups.

	Definite Vestibular Migraine (dVM) *n* = 19	Migraine (MO) *n* = 22	Healthy Controls (HC) *n* = 63	*p*-Value	*Post-hoc* Test (Tukey)	
					Comparison	*p*-Value
Physical functioning (PF, min = 1, max = 3)	2.34 (0.279)	2.61 (0.432)	2.86 (0.328)	**<0.0001**	VM-MO	**0.026**
					VM-HC	**<0.0001**
					MO-HC	**0.017**
Role-Physical (RF, min = 1, max = 2)	1.18 (0.201)	1.43 (0.431)	1.83 (0.308)	**<0.0001**	VM-MO	**0.039**
					VM-HC	**<0.0001**
					MO-HC	**<0.0001**
					MO-HC	0.048
Bodily Pain (BP, min = 1, max = 5.5)	2.61 (0.542)	3.52 (0.970)	4.65 (0.883)	**<0.0001**	VM-MO	**0.002**
					VM-HC	**<0.0001**
					MO-HC	**<0.0001**
General Health (GH, min = 1, max = 5)	2.31 (0.555)	3.35 (0.851)	3.92 (0.655)	**<0.0001**	VM-MO	**<0.0001**
					VM-HC	**<0.0001**
					MO-HC	**0.004**
Vitality (VT, min = 1, max = 6)	2.91 (0.630)	3.69 (0.626)	4.03 (0.779)	**<0.0001**	VM-MO	**0.001**
					VM-HC	**<0.0001**
					MO-HC	0.082
Social Functioning (SF, min = 1, max = 5)	2.76 (0.562)	3.66 (0.714)	4.29 (0.749)	**<0.0001**	VM-MO	**0.0002**
					VM-HC	**<0.0001**
					MO-HC	**0.0021**
Role-Emotional (RE, min = 1, max = 2)	1.14 (0.231)	1.62 (0.415)	1.78 (0.369)	**<0.0001**	VM-MO	**0.0001**
					VM-HC	**<0.0001**
					MO-HC	0.155
Mental Health (MH, min = 1, max = 6)	3.76 (0.536)	4.15 (0.401)	4.28 (0.454)	**<0.0001**	VM-MO	**0.011**
					VM-HC	**<0.0001**
					MO-HC	0.325
Health Transition (HT, min = 1, max = 5)	2.05 (0.405)	3.32 (0.995)	3.35 (0.889)	**<0.0001**	VM-MO	**<0.0001**
					VM-HC	**<0.0001**
					MO-HC	0.773

Variables are represented as mean (SD). Items are recoded so that high scores indicate good health. *p*-values are derived from the linear regression controlled for the confounding effect of age. *p*-values marked in bold indicate statistically significant differences between the groups. SF-36, The Short Form Health Survey.

**Table 3:** Association between HADS, DHI, and SF-36 subscales in the HC, VM, and MO groups.

Scale	PF	RP	BP	GH	VT	SF	RE	MH	HT	DHI
HC										
HADS	0.02 (0.920)	**−0.376 (** **0.005)**	**−0.396 (** **0.003)**	**−0.473 (<0.001)**	**−0.512 (<0.001)**	**−0.515 (<0.001)**	**−0.275 (** **0.043)**	**−0.313 (** **0.020)**	−0.248 (0.072)	**0.437 (<0.001)**
HADS-A	0.004 (0.987)	−0.22 (0.126)	**−0.401 (** **0.003)**	**−0.433 (** **0.001)**	**−0.466 (<0.001)**	**−0.464 (<0.001)**	−0.211 (0.129)	**−0.356 (** **0.007)**	−0.222 (0.112)	**0.391 (** **0.003)**
HADS-D	0.032 (0.882)	**−0.426 (0.001)**	−0.27 (0.051)	**−0.372 (** **0.004)**	**−0.406 (** **0.002)**	**−0.414 (** **0.001)**	−0.258 (0.055)	−0.173 (0.218)	−0.216 (0.12)	**0.354 (** **0.006)**
DHI	−0.124 (0.395)	−0.253 (0.078)	**−0.471 (<0.001)**	**−0.395 (** **0.003)**	**−0.372 (** **0.006)**	**−0.508 (<0.001)**	**−0.415 (** **0.002)**	**−0.388 (** **0.004)**	**−0.384 (** **0.004)**	-
dVM										
HADS	−0.384 (0.185)	−0.292 (0.332)	−0.085 (0.764)	−0.401 (0.145)	−0.267 (0.332)	−0.143 (0.575)	−0.295 (0.281)	−0.281 (0.291)	0.198 (0.471)	0.467 (0.065)
HADS-A	−0.359 (0.242)	−0.15 (0.616)	−0.263 (0.412)	−0.215 (0.456)	−0.227 (0.442)	−0.197 (0.483)	−0.337 (0.262)	−0.21 (0.456)	0.181 (0.543)	0.493 (0.074)
HADS-D	−0.361 (0.213)	−0.366 (0.241)	0.056 (0.856)	−0.496 (0.053)	−0.268 (0.332)	−0.087 (0.740)	−0.232 (0.387)	−0.303 (0.263)	0.188 (0.485)	0.397 (0.145)
DHI	**−0.862 (<0.001)**	−0.529 (0.185)	−0.62 (0.088)	−0.366 (0.290)	−0.463 (0.200)	−0.649 (0.050)	−0.389 (0.262)	−0.391 (0.262)	0.027 (0.924)	-
MO										
HADS	−0.218 (0.475)	**−0.523 (0.040)**	−0.465 (0.081)	**−0.773 (0.019)**	−0.581 (0.003)	**−0.528 (0.030)**	−0.184 (0.531)	−0.471 (0.081)	−0.12 (0.664)	0.337 (0.253)
HADS-A	−0.165 (0.572)	−0.422 (0.122)	−0.47 (0.081)	**−0.679 (0.040)**	−0.375 (0.086)	−0.461 (0.082)	−0.292 (0.365)	**−0.555 (0.039)**	−0.008 (0.971)	0.334 (0.268)
HADS-D	−0.222 (0.45)	**−0.509 (0.040)**	−0.364 (0.173)	**−0.7 (0.030)**	**−0.651 (<0.001)**	−0.481 (0.057)	−0.046 (0.881)	−0.295 (0.300)	−0.198 (0.499)	0.27 (0.368)
DHI	−0.015 (0.961)	−0.463 (0.09)	−0.212 (0.499)	−0.152 (0.671)	−0.193 (0.401)	−0.247 (0.433)	−0.498 (0.081)	−0.205 (0.506)	0.146 (0.623)	-

Effect size estimates of the associations as evaluated by regression analyses adjusted for age and sex. Standardized coefficients (β) are reported along with their corresponding *p*-values, adjusted for multiple comparisons using the Benjamini–Hochberg procedure in each group. *p*-values marked in bold indicate statistically significant associations. Significant associations are marked in bold. BP, Bodily Pain; DHI, The Dizziness Handicap Inventory; dVM, definite vestibular migraine patients; GH, General health; HADS, Hospital Anxiety and Depression Scale; HC, Healthy controls; HT, Health Transition; MH, Mental Health; MO, patients with migraine without vertigo; PF, Physical functioning; RE, Role-Emotional; RP, Role-Physical; SF, Social Functioning; VT, Vitality.

## Data Availability

The data presented in this study are available upon reasonable request from the corresponding author.

## Supplementary Materials

The following supporting information can be downloaded at: https://www.mdpi.com/article/10.3390/audiolres14050065/s1, Supplementary File S1: Informed consent form; Supplementary File S2: Checklist for patients in an acute vestibular migraine attack; Supplementary File S3: STROBE checklist.
