# Supplementary material for: Psychiatric Comorbidities and Quality of Life in Patients with Vestibular Migraine and Migraine without Vertigo: A Cross-Sectional Study from a Tertiary Clinic
Source: Audiol Res. 2024 Sep 5;14(5):778–89. doi: 10.3390/audiolres14050065 (PMC11417936; doi:10.3390/audiolres14050065)
Supplement: Supplementary file 1 [file audiolres-14-00065-s001.zip › Supplementary File S1.pdf]

Supplementary File S1

The text of the informed consent document for respondents who express a willingness to participate.

MAIN RESEARCHERS AND INSTITUTIONS:

This study is conducted within a doctoral dissertation of Franko Batinović, MD, Department of Otorhinolaryngology, University Hospital of Split, Spinčićeva 1, 21000 Split, Croatia.

Phone: +385-7-719-5505; E-mail: fbatinovic1@gmail.com

The principal investigator is Prof. Zoran Đogaš, MD, PhD, Department of Neuroscience and Sleep Medicine Center, School of Medicine, University of Split, Šoltanska 2, 21 000 Split, Croatia

Phone: +385-21-557-905; E-mail: zdogas@mefst.hr

Corresponding author: Nikolina Pleić, Department of Biology and Human Genetics, School of Medicine, University of Split, Šoltanska 2, 21000 Split, Croatia. Email: npleic@mefst.hr

INFORMED CONSENT TO PARTICIPATE IN THE RESEARCH

DURATION OF THE RESEARCH: 12 months.

PLACE OF STUDIES:

- Department of Otorhinolaryngology, University Hospital of Split, Spinčićeva 1, 21000 Split, Croatia
- Clinical Department of Diagnostic and Interventional Radiology, University Hospital Center Split

INVITATION:

Dear respondents, we invite you to participate in a scientific study that will investigate oto-neurological specifics and psychiatric comorbidities in patients with definite vestibular migraine (dVM). In our research we want to compare the Hospital Anxiety and Depression Scale (HADS), the Dizziness Handicap Inventory (DHI) scale, and the 36-item Short Form Health Survey (SF-36) scores and their subscales between dVM patients, migraine patients without vertigo (MO), and healthy controls (HCs). In the acute phase of vertigo, an otorhinolaryngologist and neurologist will examine your medical history, oto-neurological status, and investigate your hearing and balance status. Also, the psychiatrist will analyze your mental health through anamnesis, clinical status, and validated psychiatric questionnaires. Moreover, we will do *magnetic resonance imaging* of your brain to exclude central pathology. This notice will provide information designed to help you decide if you want to participate in this scientific research. Before you decide, we want you to understand why this research is being conducted and what it involves. Therefore, please read this notice carefully. If you do not understand some of the words in this notice, you can ask the doctors and members of the research team involved in this scientific research about them.

PREVIOUS KNOWLEDGE ABOUT THIS PROBLEM:

Migraine is the primary headache that affects 18% of women and 6% of men worldwide, with the highest incidence between the ages of 25 and 44. Migraine is considered the third most common disorder, and the seventh most common cause of disability in the world. The prevalence of migraine in Croatia is 16%, in Europe 15%, and ranges from 12 % to 27.5%, depending on the country. It is most often divided into migraine without aura (classic migraine) and migraine with aura. The newer classification of migraines (from 2012 and 2018) introduces a VM, which is the most common episodic vertigo in neurology and occurs in 1% of the world's adult population. The cause of VM is still unknown. The most common triggers of VM are stress, sleep deprivation, strong light, noise, and weather changes, and there is a common genetic predisposition in the background. VM can last from 5 minutes to 72 hours and greatly impair mental health. Several studies have suspected that patients with VM have poor life quality and psychiatric comorbidities. So far, the connection between the VM and psychiatric comorbidity has been poorly studied. Therefore, our goal is to determine whether or not patients with dVM have psychiatric comorbidities and what those comorbidities are.

THE HYPOTHESES OF OUR RESEARCH ARE:

- dVM will have mostly anxiety as a psychiatric comorbidity according to psychiatric examination and HADS scores.
- dVM patients will have low life quality expressed as SF-36 results compared to HCs and MO patients.
- dVM patients have a moderate dizziness score according to the DHI questionnaire.

THE GOALS OF OUR STUDIES:

The ultimate goals of our research are to examine the quality of life and evaluate which are the most common psychiatric comorbidities in patients with dVM according to clinical-based psychiatric examination and HADS questionnaire. Also, we want to compare the SF-36, DHI, and HADS results, along with their subscales, between dVM patients, MO patients, and HCs

YOUR ROLE OF RESPONDENTS IN THIS SCIENTIFIC RESEARCH:

You will be applied to the diagnostic tests described below during this research. These tests and procedures are commonly used in medical and research settings in acute vertigo attacks. In an acute attack of VM, the patient will come to the researcher at the Ear, Nose, and Throat (ENT) Clinic, University Hospital Center (UHC) Split, where he will be acquainted with all procedures that will be performed during the 72-hour stay. The patient will be in touch with the contents and conditions of the research, and the staff who will conduct the research. It entails taking a thorough history, an oto-neurological clinical examination by an ENT and neurology specialist, a psychiatric examination, filling out validated questionnaires, and an examination of the brain, auditory and vestibular systems. These examinations are chronologically arranged and elaborated in detail below.

THE FOLLOWING PROCEDURES WILL BE PERFORMED DURING THIS RESEARCH:

1. Taking anamnestic data and clinical examination by three specialists (otorhinolaryngology, neurology, and psychiatry):
   Specialists in otorhinolaryngology, neurology, and psychiatry with more than 5 years of work experience at the UHC Split will take comprehensive anamnestic data, and perform a detailed clinical examination. A specialist in neurology and ENT will perform an oto-neurological examination, which includes: a local examination of the ear (otoscopy); examination of the function of the cerebral nerves; examination of vestibular-ocular reflex (bulbomotor test with emphasis on spontaneous, provocative, and positional nystagmus, smooth following and saccades, positional tests, cover test, head impulse test, head-shaking test), vestibulospinal and vestibulocerebellar reflex arc (walking tests, anticipatory tests, Romberg test, etc.). This procedure typically last 20 to 30 minutes. The psychiatrist will take a psychiatric history and clinical examination according to the Diagnostic and Statistical Manual 5 of Mental Disorders criteria to determine the existence of psychiatric disorders such as anxiety, depression, phobic disorders, etc.
2. Recording demographic and anthropometric data:

   Demographic characteristics of all VM patients will be collected and included: age (year), gender (M/F), residence (town/village), and marital status (single; married; divorced; widowed; separated). Also, anthropometric data, including height (cm), weight (kg), and body mass index (BMI), will be noted.

3. Questionnaires survey:

In the symptom-free period, all participants need to complete the HADS, SF-36, and the DHI questionnaires.

- Hospital Anxiety and Depression Scale (HADS)

This is a fourteen-item scale with seven sections for anxiety and depression subscales. We will use it because we expect psychiatric comorbidity in VM patients, such as anxiety, depression, functional disorders, and phobic disorders. All dVM patients will be asked to complete the HADS with the assistance of a psychiatrist within 72 hours of coming to EED. Before patients answer the questions, the psychiatrist will confirm that the individual understood them all. Otherwise, the psychiatrist will clarify it for them. A single interview lasted about 10 - 15 minutes. The HADS will be distributed in Croatian.

- Dizziness Handicap Inventory Scale (DHI)

DHI scale consists of 25 questions that can be used to assess the extent of the impairment in patients with vertigo based on emotional, functional, and physical aspects. Patients may have minor, moderate, and severe disorders according to the questionnaire.

- Short Form Health Survey (SF-36)

The SF-36 includes 36 questions and eight health domains divided into two dimensions: physical health and mental health. "Physical functioning", "Role limitations due to physical health", "Pain", and "General health" domains comprise the physical health dimension of patient quality of life, whereas "Energy/fatigue", "Social functioning", "Role limitations due to emotional problems", and "Emotional wellbeing" domains comprise the mental health dimension. From the perspective of the patients the SF-36 will assess disease burden. It takes about five to ten minutes to complete.

4. The brain MRI:

All patients will take a 1,5 Tesla magnetic brain scan at the Clinical Department of Diagnostic and Interventional Radiology, UHC Split to rule out other central neurological disorders. The patient must lie flat on his back and he will hear a loud sound, but don't be afraid. It is important to know that the patient can cancel the scan at any time. The magnetic brain scan takes about 20 minutes.

HOW MUCH WILL I PARTICIPATE IN THIS RESEARCH?

The VM patients will participate in the study from 24 to 72 hours. MO patients will participate for 2 hours, and HCs will participate for 30 minutes. Participation for VM patients involves taking a comprehensive medical history with anthropometric parameters, audio-vestibular diagnostic tests, neurological examination, psychiatric examination, recording MRI of the brain, and completing a questionnaire (DHI, SF-35, and HADS).

WHAT ARE THE POSSIBLE BENEFITS OF PARTICIPATION FOR RESPONDENTS?

There is no guarantee that you will benefit from participating in the research. If you agree to participate in this research, we guarantee you an individualized approach to your vertigo, migraine, and mental disorders. It involves a detailed examination of your neurological and balance system, as well as your mental health. With this research, you have a unique opportunity to test the functionality of your

hearing and balance system. All this will be analyzed by a multidisciplinary scientific team consisting of otorhinolaryngologists, neurologists, psychiatrists, and radiologists. The ultimate benefit of this research is the potential to detect some psychological problems. During the research, you will be provided with an insight into all new findings, as well as all relevant findings of the performed searches.

WHAT ARE THE POSSIBLE RISKS OF PARTICIPATING IN THIS RESEARCH?

Examination of auditory and vestibular function: there are no significant risks and dangers when examining vestibular function by performing a *Dix Hallpike* positional test, measuring *subjective visual vertical* or *video head impulse test* (VHIT), as well as recording audiometry in an adult diagnosed with VM. These diagnostic tests are standardized methods that serve to distinguish VM from other oto-neurological disorders. There is a possibility of transient nausea when performing the Dix Hallpike positional test and VHIT.

MRI of the brain: in the symptom-free phases of your disorder you will have an MRI of the brain. It takes 15 to 30 minutes and there is the possibility of transient noise and discomfort produced by the machine itself when recording your brain. It is a non-invasive and without-radiation diagnostic tool and results will be read by a radiology specialist.

MUST PARTICIPATE?

It is up to you to freely decide whether you want to participate in this research or not. If you choose to participate, you will receive this notice to sign (keep one copy). Your participation is voluntary, and you can withdraw freely and without any consequences at any time, without giving a reason. You will continue to be treated in the way that is usual for your illness. If you decide to discontinue participation, please inform the principal investigators and their collaborators on time.

CONFIDENTIALITY AND INSPECTION OF DOCUMENTATION

The introductory part of the interview will emphasize the confidentiality of information and the content of interviews between respondents and researchers. By giving informed consent, respondents sign that they are familiar with the objectives of the research and agree to participate in the research. Your data will be entered electronically (in the hospital information system called BIS) as any patient who comes to the Emergency ENT Department with acute vertigo or headache. Principal investigators and their collaborators will adhere to internal procedures for the protection of personal data. You will be entered into the database (excel tables) using the code name of this survey (OSCAR 1, OSCAR 2, etc.). Your medical records will be reviewed by principal investigators and their collaborators. Representatives of the Ethics Committee of the UHC Split can also have access to the documentation.

WHAT WILL THE DATA OBTAINED IN THIS SCIENTIFIC RESEARCH BE USED FOR?

Data from this scientific research can be of practical use, but also scientific, and will be published in scientific publications. Your identity in the *BIS* will be visible to other doctors at the UHC Split, but it will remain anonymous in our database.

WHO ORGANIZES AND FINANCES THE RESEARCH?

The Otorhinolaryngology Department of the UHC Split organizes the examination. Also, cooperation between the ENT, Neurology, Psychiatry, and Radiology Clinics was of utmost importance. There was no additional funding for our research.

WHO REVIEWED THIS TRIAL?

This study was reviewed by the Ethics Committees of the Medical Faculty of the University of Split and the UHC Split, which approved this research after reviewing the relevant documentation. The study is conducted according to all applicable guidelines, which aim to ensure the proper conduct and safety of the persons participating in this scientific research, including the Fundamentals of Good Clinical Practice and the Declaration of Helsinki.

WHOM TO CONTACT FOR FURTHER INFORMATION?

If you need additional information, feel free to contact the principal investigators. Any respondent may at any time request the deletion of personal data from the research medical report by sending a simple request to the study coordinator, Franko Batinović MD, (e-mail contact: fbatinovic@kbsplit.hr). If respondents do not request the withdrawal from the study, we would like to emphasize that published, anonymized research results cannot be removed (e.g., research results are published in a scientific journal). To promote free access to scientific information and avoid unnecessary research, anonymous transcribed data from conducted examinations will be available in the OSF register (Open Science Framework, https://osf.io/). Anonymity will be ensured by removing all names.

VIOLATION OF DATA CONFIDENTIALITY

In the event of a data breach, we will contact the affected respondents and the data will be temporarily removed from the compromised storage.

INFORMED CONSENT

Participation in this study is voluntary, and respondents can withdraw from the study at any time without giving reasons for withdrawal. If you want to withdraw from the study, please contact the principal investigator, Franko Batinović (fbatinovic@kbsplit.hr).

- I have read information about the study. I had the opportunity to ask questions and get satisfactory answers. I had enough time to decide whether to participate in this study.
- I am aware that my participation in the study is voluntary. Also, I know that I can choose not to participate or withdraw from this study at any time. I am not obliged to state the reasons for non-participation or withdrawal from the study.
- I give my permission for my examination data and collected blood can be used for scientific purposes.
- I consent to my anonymized and transcribed examination data being publicly available on the OSF scientific platform. I understand that this means that my anonymized data may be used for research purposes other than those described above. I am also aware that this means that my anonymized data may be used in countries outside Europe and that data use regulations may be different from those in the European Union.

- I understand that the responsible individuals like the main researchers and their associates, and the members of the UHC Split have access to my medical documentation. I permit these individuals to access my medical records.
- I agree that my family doctor (or family member) be aware of my participation in the above scientific research.
- I want to participate in this study.

Respondent:

Name and surname (in block letters): ____

Date: ____

Signature:____

The person who conducted the notification procedure for the respondent and the consent to participate:

Name and surname (in block letters): ____

Date: ____

Signature:____

Principal Investigator of the project:

Name and surname (in block letters): ____

Date: ____

Signature:____
