# Supplementary material for: Psychiatric Comorbidities and Quality of Life in Patients with Vestibular Migraine and Migraine without Vertigo: A Cross-Sectional Study from a Tertiary Clinic
Source: Audiol Res. 2024 Sep 5;14(5):778–89. doi: 10.3390/audiolres14050065 (PMC11417936; doi:10.3390/audiolres14050065)
Supplement: Supplementary file 1 [file audiolres-14-00065-s001.zip › Supplementary File S2.pdf]

Supplementary File S2

Doctor's questions for patients in an acute vestibular migraine attack.

MAIN RESEARCHERS AND INSTITUTIONS:

This study is conducted within a doctoral dissertation of Franko Batinović, MD, Department of Otorhinolaryngology, University Hospital of Split, Spinčićeva 1, 21000 Split, Croatia.

Phone: +385-7-719-5505; E-mail: fbatinovic1@gmail.com

The principal investigator is Prof. Zoran Đogaš, MD, PhD, Department of Neuroscience and Sleep Medicine Center, School of Medicine, University of Split, Šoltanska 2, 21 000 Split, Croatia

Phone: +385-21-557-905; E-mail: zdogas@mefst.hr

Corresponding author: Nikolina Pleić, Department of Biology and Human Genetics, School of Medicine, University of Split, Šoltanska 2, 21000 Split, Croatia. Email: npleic@mefst.hr

CHECKLIST

Name and surname_____________________________; Age_______________________

There can be one or multiple answers.

1. The vestibular symptoms occurred in the form of:

- Episodic spontaneous vertigo
- Episodic positional vertigo
- Episodic spontaneous and positional vertigo

2. VM attack was accompanied by:

- Nausea without vomiting
- Nausea with vomiting
- Light sensitivity
- Sound sensitivity
- Odor sensitivity
- Nothing specified

3. Do the attacks limit your physical activity?

- Yes
- No

4. Trigger(s) of the attack:

- The attack occurred spontaneously
- The attack happened after a stressful event
- The attack was preceded by changes in weather conditions
- Poor sleep immediately before the attack
- The attack was preceded by a sudden head movement
- Exposed to strong visual or auditory stimuli immediately before the attack
- Prolonged use of computer before the attack
- Prolonged use of a mobile phone before the attack

5. How many attacks of migraine and vertigo have you experienced so far in your life?

- Up to 5 attacks
- 5 to 10 attacks
- 10 to 15 attacks
- 15 to 20 attacks
- More than 20 attacks

6. The frequency of vestibular migraine attacks is:

- 2 or more times per week
- Once a week
- Once a month
- 1 to 2 times a year
- 3 to 6 times a year

7. Vestibular migraine attacks most commonly occur:

- In the morning (12 a.m. to 6 a.m.)
- From 6 a.m. to 12 p.m.
- From 12 p.m. to 7 p.m.
- From 7 p.m. to 12 a.m.

8. Typically, the duration of a vestibular migraine attack is:

- From 20 minutes to 60 minutes
- From 1 hour to 6 hours
- From 6 hours to 24 hours
- From 24 hours to 48 hours

- From 48 hours to 72 hours
- Longer than 72 hours

9. How long have you been experiencing vestibular migraine attacks?

- Less than 5 years
- 5-10 years
- 10-15 years
- More than 15 years

10. Vestibular migraine attacks usually resolve after:

- Taking medication (specify the medication) _______________________
- Sleeping
- Withdrawing to a quiet and dark room
- Showering with cold water
- Spontaneously

11. Do you have any other ENT or neurological conditions?

- Yes (Specify the diagnosis): ________________________________
- No

12. Headache:

- Does not experience headaches
- Headache precedes the dizziness
- Headache occurs after experiencing dizziness
- Headache occurs during dizziness

13. The headache is:

- Right-sided hemicephalic
- Left-sided hemicephalic
- Bilateral
- Not present

14. The headache is located:

- Occipital
- Frontal
- Behind the eye/temporal

- In the neck area
- Not present

15. The character of the headache:

- Tightening
- Pulsating
- Dull
- Stabbing
- None

16. Aura:

- Not present
- Aura preceded the mentioned headache and dizziness, lasting less than 60 minutes
- Aura preceded the mentioned headache and dizziness, lasting more than 60 minutes
- Aura was visual (visual field loss, sparks, flickering lights, dots, etc.)
- Somatosensory (tingling and burning sensations on the face)
- Motor (weakness in limbs)

17. How long have you been experiencing migraine?

- Less than 5 years
- 5-10 years
- 10-15 years
- More than 15 years
- Not present

18. Typically, a migraine headache lasts:

- From 20 minutes to 60 minutes
- From 1 hour to 6 hours
- From 6 hours to 24 hours
- From 24 hours to 48 hours
- From 48 hours to 72 hours
- Longer than 72 hours
- Not present

19. Migraine headache most commonly occurs:

- In the morning from 12 am to 6 am

- From 6 am to 12 pm
- From 12 pm to 7 pm
- From 7 pm to 12 am
- Not present

20. Migraine headache most commonly occurs:

- In the morning from 12 am to 6 am
- From 6 am to 12 pm
- From 12 pm to 7 pm
- From 7 pm to 12 am
- Not present

21. Did you use medications during an acute attack at home:

- Did not use medication to alleviate the acute attack
- Used medication to alleviate the acute attack (name and dosage):

  ____

22. Hearing sensations in acute vestibular migraine attack:

- During the attack, hearing in both ears was normal
- During the attack, hearing was impaired in one ear
- During the attack, the hearing was impaired in both ears

23. Tinnitus:

- Does not complain of tonal noise
- Complains of tonal noise in both ears
- Complains of tonal noise in the right ear
- Complains of tonal noise in the left ear
- Does not complain of pulsating noise
- Complains of pulsating noise in both ears
- Complains of pulsating noise in the right ear
- Complains of pulsating noise in the left ear

24. Fullnes of ears:

- Denies feeling of ear fullness
- Complains of the feeling of ear fullness

25. Circle only one answer:

Frequent morning nausea: YES / NO

Susceptible to motion sickness: YES / NO

Sensitive to changes in weather: YES / NO

Complains of frequent neck pain: YES / NO

Complains of frequent visual fatigue and/or visual hypersensitivity: YES / NO

Complains of frequent osmophobia: YES / NO

Complains of frequent phonophobia: YES / NO

Experiences headaches during menstruation: YES / NO / Male

26. Associated discomfort:

- The attack is not accompanied by discomfort such as sweating, shortness of breath, palpitations, tingling in the body/face, shaking, thirst, weakness, diarrhea, paleness, discomfort/pressure in the chest, abdominal discomfort, fear, fear of losing control, feeling cold, hot flashes, feeling of derealization, feeling of detachment.
- The attack is accompanied by discomfort such as sweating, shortness of breath, palpitations, tingling in the body/face, shaking, thirst, weakness, diarrhea, paleness, discomfort/pressure in the chest, abdominal discomfort, fear, fear of losing control, feeling cold, hot flashes, feeling of derealization, feeling of detachment.

27. Having omnious neurologic "D" symptoms:

- Denies loss of consciousness, loss of vision, double vision, hoarseness, difficulty swallowing, difficulty speaking, tingling and weakness in the face and limbs, sensory loss.
- Reports the presence of central symptoms, such as ________________________

28. Habits:

- Normal
- Smoking cigarettes, ____ cigarettes/day / ____ years
- Drinking coffee, _______ ml per day
- Consume alcohol, _________ ml per day

29. Family history:

- Close family members suffer from headaches
- Close family members do not suffer from headaches
- Close family members suffer from headaches and dizziness
- Denies that close family members suffer from headaches and dizziness

30. Circle the bolded expressions (from 1 to 8) that best describe your current symptoms (there can be multiple):

1. Spontaneous vertigo is a sensation of spinning of the surrounding space without an obvious trigger, and you would describe it as: spinning, swaying, tilting, rocking, swaying, falling, floating?

2. Discomfort with movement and standing - any loss of balance without a sense of space rotation.

3. Visually induced vertigo - vertigo induced by complex or distorted visual stimuli that occupy a significant portion of the visual field or moving visual stimuli, including relative motion of the visual environment associated with one's own movement.

4. Head motion-induced vertigo - vertigo that occurs only during head movements.

5. Positional vertigo - vertigo that occurs with changes in head or body position in space.

6. Oscillopsia - a sensation of visual image flickering.

7. Visual lag - the illusion of the visual environment lagging behind after a rapid head movement.

8. Postural symptoms - balance symptoms related to the sustainability of an upright posture (sitting, standing, or in motion); this includes a feeling of instability and a need to hold on when standing and a feeling of falling (tendency to fall or veer in a specific direction).

Number of current vestibular symptoms: _________________
